# The anti-inflammatory drugs diclofenac and ketorolac inhibit urease activity and biofilm formation by uropathogenic *Proteus mirabilis*

**DOI:** 10.1007/s12223-025-01336-7

**Published:** 2025-09-22

**Authors:** Afaf Eladl, Hisham A. Abbas, Samar S. Elbaramawi, Marwa Ibrahim Abd El-Hamid, Shaimaa I. Nazeih

**Affiliations:** 1 Department of Microbiology and Immunology, Faculty of Pharmacy, Zagazig University, Zagazig, 44519 Egypt; 2 Department of Medicinal Chemistry, Faculty of Pharmacy, Zagazig University, Zagazig, 44519 Egypt; 3 Department of Microbiology, Faculty of Veterinary Medicine, Zagazig University, Zagazig, 44519 Egypt

**Keywords:** *Proteus mirabilis*, Urease inhibition, Chelation, Diclofenac sodium, Ketorolac tromethamine, CAUTI

## Abstract

*Proteus mirabilis* is a major bacterium responsible for catheter-associated urinary tract infections (CAUTIs). Urease enzyme has a great role in the pathogenesis of *Proteus mirabilis.* Using urease,* Proteus mirabilis* can decompose urea to produce ammonia that increases urine pH and enhances crystal precipitation and crystalline biofilm formation. This leads to catheter blockage and pyelonephritis, respectively. Urease inhibitors are of great value in controlling this problem. Diclofenac sodium and ketorolac tromethamine, with their metal chelating activities, can inactivate urease by chelation of nickel ion in the active site of urease. This study investigated the ability of diclofenac sodium and ketorolac tromethamine to inhibit urease activity in* Proteus mirabilis* and crystalline biofilm formation. Diclofenac sodium and ketorolac tromethamine showed comparable activities against urease in cell lysates and whole cultures, with subsequent inhibition of pH increase and crystal formation in artificial urine. Diclofenac sodium showed higher biofilm inhibition and downregulation of urease genes *ureR and ureC* in RT-qPCR*.* The docking study showed the ability of both drugs to bind to urease enzyme and to chelate nickel ions in the active site of urease, suggesting that nickel chelation is the mode of inhibition of urease enzyme. In conclusion, diclofenac sodium and ketorolac tromethamine are two urease inhibitors that may be useful in treating *Proteus mirabilis* CAUTI*.*

## Introduction

*Proteus mirabilis* is a Gram-negative bacillus and a frequent causative agent of catheter-associated urinary tract infections (CAUTIs) in hospitalized patients (Armbruster et al. 2017, 2018). The infection caused by *Proteus mirabilis* is related to its ability to secrete urease enzyme. Urease cleaves urea present in urine to ammonia and carbon dioxide. Ammonia makes the urine alkaline to precipitate crystals in catheters that may be blocked by formation of crystalline biofilms inside the lumina of the catheters (Li et al. 2002; Stickler and Feneley 2010; Stickler 2014; Schaffer et al. 2016). *Proteus mirabilis* was reported in 80% of blocked catheters, so it represents the most frequent ureolytic bacteria that are related to catheter blockage (Nicolle 2014). When blocked, the urinary catheter causes painful bladder distension and urine reflux to the kidneys, leading to increased risk of pyelonephritis, in addition to bloodstream infection, urosepsis, and increased mortality (Hooton et al. 2010).

The management of catheter blockage depends on replacement by a new one or antibiotic therapy. On removing the blocked catheter, the patient feels pain. Moreover, the catheter is anchored to the bladder due to encrustation and the urethra may be damaged during the removal of the catheter **(**Hooton et al. 2010**).** Using antibiotics, on the other hand, is not the optimum solution due to the development of antibiotic resistance (Niël-Weise and van den Broek, 2005). As a result, new therapeutic options are urgently needed. One of these options is the use of urease inhibitors. Inhibiting urease can prevent the increase in urinary pH and precipitation of urinary stones that block catheters and the formation of biofilms inside the catheters (Milo et al. 2021). Urease is a metalloenzyme that has nickel ions in its active site. Chelation of nickel ions can inhibit urease activity (Krajewska 2009; Modolo et al. 2015).

Diclofenac and ketorolac are two anti-inflammatory drugs. Diclofenac is found to have anti-virulence activities against some bacteria such as *Pseudomonas aeruginosa* and *Staphylococcus aureus* (Abbas 2015; Abbas et al. 2020). Moreover, ketorolac synergizes with gentamicin against planktonic and biofilm cells of *Staphylococcus aureus* (Sekar et al. 2024). It is known that several derivatives of non-steroidal anti-inflammatory drugs (NSAID) act via chelation to inhibit the activity of metalloenzymes. Diclofenac can form diclofenac-metal complexes with copper, nickel, manganese, and cobalt that are found to have good anti-inflammatory activities (Kovala-Demertzi et al. 1998).

In this study, the inhibition of urease enzyme produced by uropathogenic *Proteus mirabilis* by diclofenac and ketorolac was investigated.

## Materials and methods

### Bacterial isolates and chemicals

The bacterial strain used in this study is a clinical isolate of *Proteus mirabilis* that was originally isolated from a patient with CAUTI. It was provided by the Microbiology and Immunology Department, Faculty of Pharmacy, Zagazig University. Diclofenac sodium was supplied by Novartis, Egypt, and ketorolac tromethamine was purchased from Amriya pharmaceuticals, Egypt. Dimethyl sulphoxide (DMSO) was purchased from Sigma Chemical Co. (St. Louis, MO, USA). All media were purchased from Oxoid, UK.

### Determination of the minimum inhibitory concentration (MIC) of diclofenac sodium and ketorolac tromethamine against Proteus mirabilis

The minimum inhibitory concentrations (MICs) of diclofenac sodium and ketorolac tromethamine were determined using the broth microdilution method (Wayne 2011). Briefly, 1% of *P. mirabilis* culture (10^8^ CFU/ml) was prepared and delivered as aliquots (50 μl) to the wells of a microtiter plate that were supplemented with equal volumes of twofold serial dilutions of diclofenac sodium and ketorolac tromethamine. The plate was incubated at 37 °C for 24 h, and the minimum inhibitory concentration (MIC) was calculated as the concentration resulting in complete inhibition of visible growth.

### Effect of the sub-inhibitory concentration of diclofenac sodium and ketorolac tromethamine on bacterial growth

In order to ensure that the used sub-inhibitory concentration of diclofenac sodium and ketorolac tromethamine had no effect on bacterial growth, overnight cultures of control *Proteus mirabilis* and treated ones with sub-inhibitory concentrations of diclofenac sodium and ketorolac tromethamine (¼ MIC equal to 2.5 mg/ml) were prepared. The turbidities of the resultant overnight cultures were measured at 600 nm using a UV–Vis microplate reader (synergy HT BioTek) and compared (Koch 1970).

### Urease inhibition assay

The potential ability of diclofenac sodium and ketorolac tromethamine to inhibit urease activity was assessed (Durgadevi et al. 2019) using 2.5 mg/ml of each drug. For the whole cell assay, *P. mirabilis* culture (1%) was added to 1 ml of urea broth that contained peptone (0.15%), sodium chloride (0.5%), potassium dihydrogen phosphate (0.2%), and urea (2.0%, sterilized by filtration) with and without the tested drugs for 24 h at 37 °C. Then, the cultures were centrifuged, and phenol red reagent (0.02%) was added as a pH indicator to the cell-free supernatants of control and treated cultures. In order to quantitate the inhibition of urease activity in cell lysate, the cultures were centrifuged, and the pellets were resuspended in 2 mL of 10 mM K_2_HPO_4_ solution followed by sonication of the bacterial cells (90 s with 0.5 cycles at 100% amplitude) in a container with ice to release urease, and phenol red reagent (0.02%) was added in the same way. Urease activity increases the pH that can be noticed by a color change from yellow to pink. Pink color was observed visually and measured by spectrophotometer by measuring absorbance at 570 nm._._ The percentage of inhibition of urease activity was determined, and pH increase was measured in the presence and absence of the tested drugs at zero time, after 2 h and 24 h.

### Inhibition of crystal formation

Aided by urease activity, *P. mirabilis* can produce crystals in artificial urine (AU) media composed of calcium chloride (0.05%), magnesium chloride (0.07%), sodium chloride (0.5%), sodium oxalate (0.002%), sodium disulfate (0.2%), trisodium citrate (0.06%), 0.3% potassium dihydrogen orthophosphate, ammonium chloride (0.1%), potassium chloride (0.2%), and urea (2.5%) dissolved in sterile distilled water with pH adjusted to 6.2 ± 0.2. AU was filter-sterilized and pooled with an equal volume of sterile 2 × LB medium. The effect of diclofenac sodium and ketorolac tromethamine (at a concentration of 2.5 mg/ml) on *P. mirabilis* crystal formation was then assessed. *P. mirabilis* culture (1%) was prepared in AU media with and without the tested drugs and incubated for 24 h at 37 °C to firm the crystals. The crystals were visualized under the light microscope (Tanaka et al. 2003).

### Assay of inhibition of biofilm formed on urinary catheters

The ability of the two drugs (at a concentration of 2.5 mg/ml) to inhibit biofilm formation on urinary catheters was assessed (Stepanovic et al. 2000). *Proteus mirabilis* was cultured in Tryptone soya broth (TSB) and incubated overnight at 37 °C. The resultant suspension was diluted to have a suspension with 10^6^ CFU/ml. Five-milliliter aliquots of the suspension were transferred to 15-mL sterile falcon tubes containing artificial urine media and pieces of urinary catheter (1 cm long). The falcon tubes may have either diclofenac sodium or ketorolac tromethamine or have no drugs (control). The tubes were incubated for 24 h at 37 °C, and the catheter pieces were removed from the tubes for washing with phosphate-buffered saline. The biofilms were fixed with methanol (99%) and stained with crystal violet (1%) before washing with distilled water. The adherent dye was solubilized with glacial acetic acid (33%), and the absorbance of eluted crystal violet was measured at 590 nm with a Biotek Spectrofluorimeter (Biotek, USA), and % of inhibition was calculated.

### Reverse transcriptase qPCR of urease genes

To investigate the anti-urease activity of diclofenac sodium and ketorolac tromethamine (at a concentration of 2.5 mg/ml) at the molecular level, reverse transcriptase qPCR was used to assess the effect of the two drugs on the expression of *UreR* and *UreC* genes that encode urease enzyme, and the levels of expression were normalized relative to the housekeeping gene *16S rRNA*. The primers are listed in Table 1. StepOne Real-Time PCR system (Applied Biosystem, USA) was used following SensiFAST™ SYBR® Hi-ROX One-Step Kit protocol (Bioline, UK). The relative gene expression was calculated by using the comparative threshold cycle (^∆∆Ct^) method (Livak and Schmittgen 2001).

**Table 1:** Primers used in RT-PCR (Zhang et al. 2013; Kafe et al. 2023)

Gene name	Primer sequence
*ureR* (F) *ureR* (R)	GGTGAGATTTGTATTAATGG ATAATCTGGAAGATGACGAG
*ureC* (F) *ureC* (R)	CCGGAACAGAAGTTGTCGCTGGA GGGCTCTCCTACCGACTTGATC

### In silico study of binding of diclofenac sodium and ketorolac tromethamine

The possible binding of diclofenac sodium and ketorolac tromethamine to urease enzyme was investigated by in silico study using UCSF Chimera 1.19 **(**Pettersen et al. 2004**)** and Autodock Vina (Trott O and Olson 2010) in Chimera. The crystal structure of *Sporosarcina pasteurii* urease (PDB: 4UBP; 1.55 Å) was downloaded from the (RCSB Protein Data Bank). The protein structure was optimized through the Chimera Dock Prep tool and saved as a PDB file.

The chemical structures of diclofenac, ketorolac, and urea were retrieved from PubChem ID in Chimera. Ligand structures were optimized through the Chimera Dock Prep tool and saved as a mol2 file. Docking procedures were performed through the Autodock Vina tool in Chimera.

### Statistical analysis

All the experimental investigations were replicated, and the mean and standard errors were calculated. One-way ANOVA statistical tests were employed to determine the significance, where *p* < 0.05 was considered significant.

## Results

### Antibacterial activity of tenoxicam against Proteus mirabilis

#### Antibacterial activity and effect of sub-inhibitory concentration of diclofenac sodium and ketorolac tromethamine on the growth of Proteus mirabilis

**Fig. 1:**
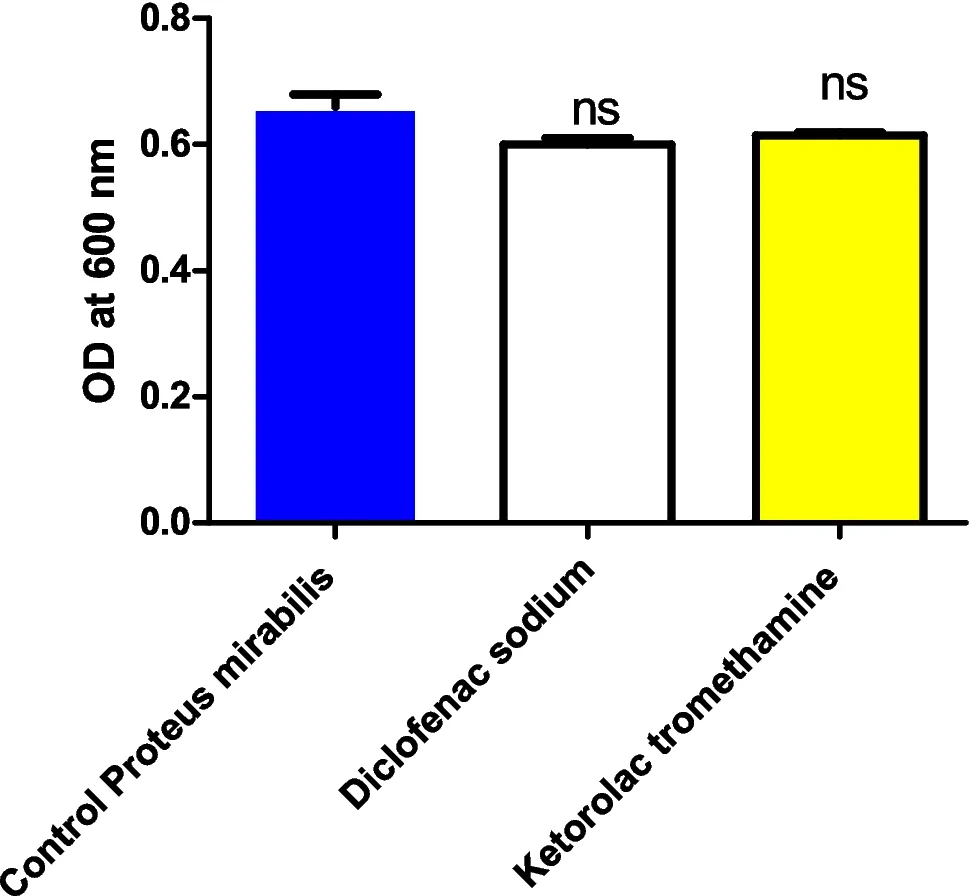
Sub-MIC of diclofenac sodium and ketorolac tromethamine had no significant effect on bacterial growth of the tested strain, *P* > 0.05; ns indicates no significant difference

The minimum inhibitory concentration of diclofenac sodium and ketorolac tromethamine against the test isolate was determined according to CLSI guidelines. They could inhibit the growth at 10 mg/ml. The effect of ¼ MIC of both drugs on the growth of *Proteus mirabilis* was investigated, and it showed no significant effect on bacterial growth, as shown in Fig. 1. This sub-MIC was used in all the next experiments to ensure that the used concentration affects urease and biofilm away from the effect on growth.

#### Diclofenac sodium and ketorolac tromethamine decreased urease activity

**Fig. 2:**
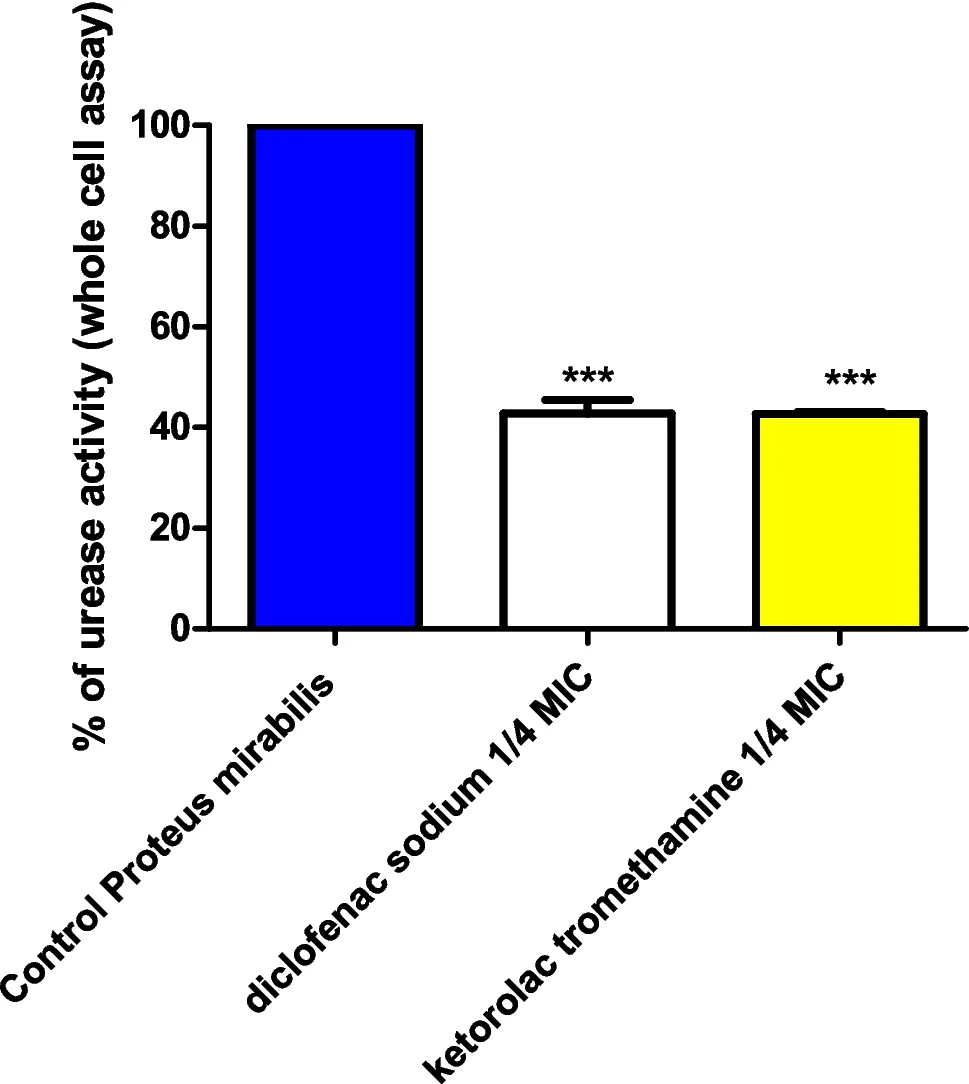
Inhibition of urease in whole cell cultures by diclofenac sodium and ketorolac tromethamine. Diclofenac sodium and ketorolac tromethamine significantly reduced urease enzyme activity, *P* < 0.05. ***Significant inhibition

In order to assess the ability of diclofenac sodium and ketorolac tromethamine to inhibit urease activity, the inhibition of color change of urea broth from yellow to pink due to urease activity and increase in pH was performed by visual inspection and measurement of the absorbance at 570 nm (Fig. 2). The two drugs showed comparable urease inhibiting activities. Diclofenac sodium decreased the urease activity from 100% in the control culture to only 42.79% in the treated culture. On the other hand, ketorolac tromethamine decreased the urease activity from 100% in the control culture to only 42.69% in the treated culture.

**Fig. 3:**
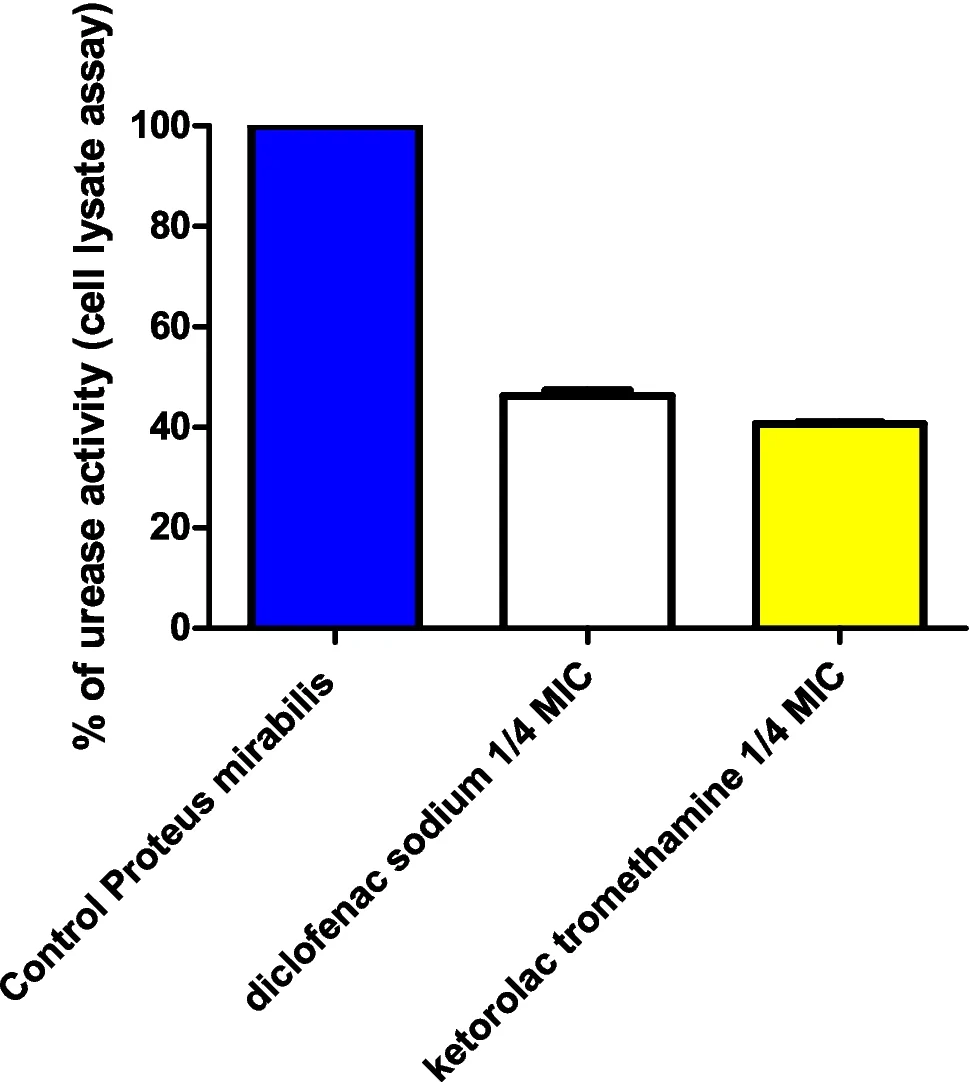
Inhibition of urease in cell lysate by diclofenac sodium and ketorolac tromethamine. Diclofenac sodium and ketorolac tromethamine significantly reduced urease enzyme activity, *P* < 0.05. ***Significant inhibition

For further confirmation, the urease activity from lysed cells was quantified in the same way as the whole cell urease. Again, the urease inhibition by the two drugs was more or less similar. Diclofenac sodium could reduce urease activity to 46.25%, while ketorolac tromethamine could reduce urease activity to 40.75% (Fig. 3).

**Fig. 4:**
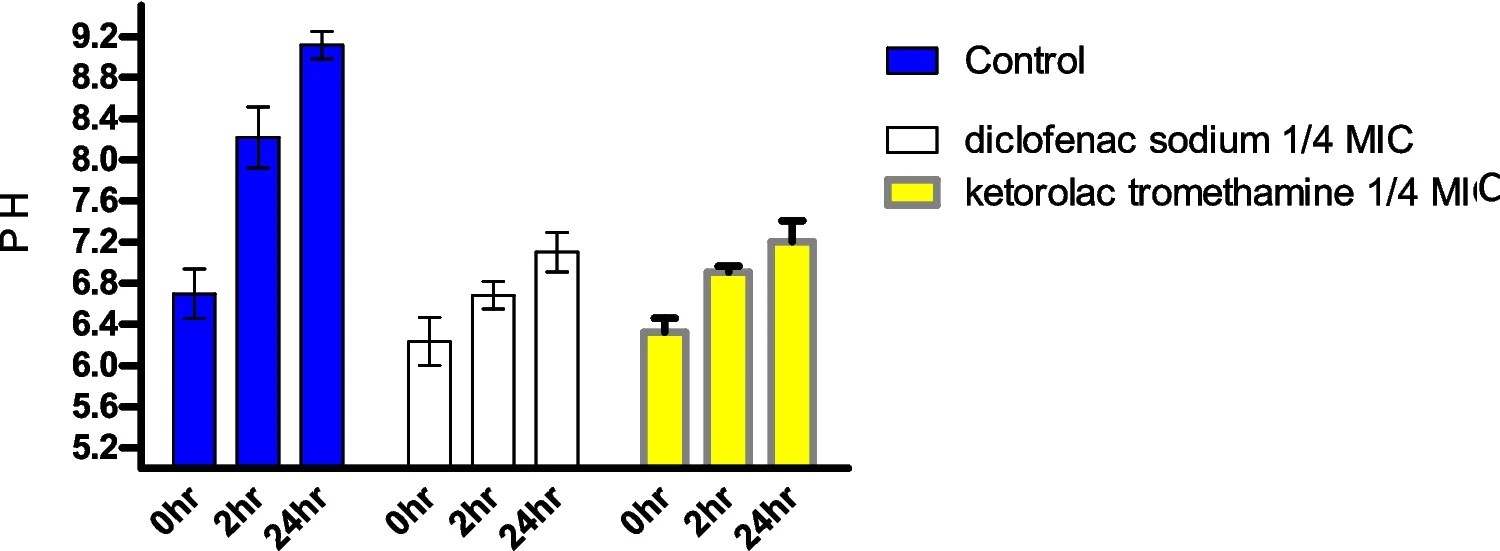
Diclofenac sodium and ketorolac tromethamine decreased the pH shift due to urease activity after 2 and 24 h

 Diclofenac sodium and ketorolac tromethamine could also reduce the pH increase due to urease activity that results in the release of ammonia (Fig. 4). In control culture, pH increased from 6.53 at the beginning of the experiments to 8.01 after 2 h and then to 9.02 after 24 h. On the other hand, in the presence of diclofenac sodium, pH increased from 6.4 at zero time to 6.78 after 2 h and then to 7.24 after 24 h, while in the presence of ketorolac tromethamine, pH increased from 6.42 at zero time to 6.95 after 2 h and then to 7.35 after 24 h, a result coincident with their effects on urease inhibition.

#### Diclofenac sodium and ketorolac tromethamine inhibited crystals formation in artificial urine

**Fig. 5:**
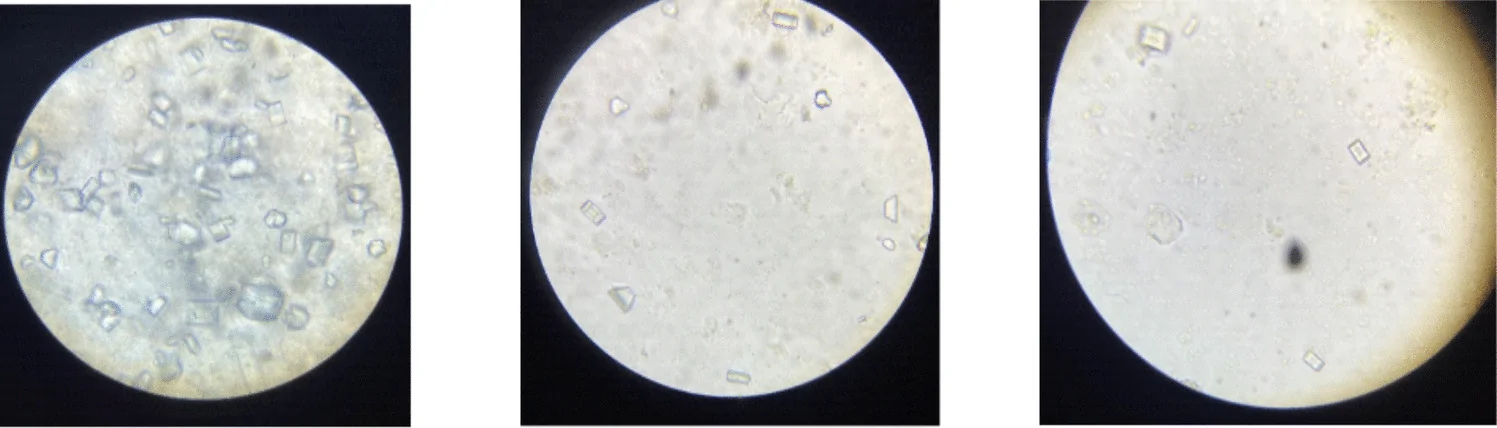
Diclofenac sodium and ketorolac tromethamine inhibited crystal formation in artificial urine

Urease enzyme degradation of urea leads to pH increase that facilitates crystal formation in urine. In this study, diclofenac sodium and ketorolac tromethamine could block crystal formation in AU (Fig. 5).

#### Diclofenac sodium and ketorolac tromethamine inhibited biofilm formation on catheter

**Fig. 6:**
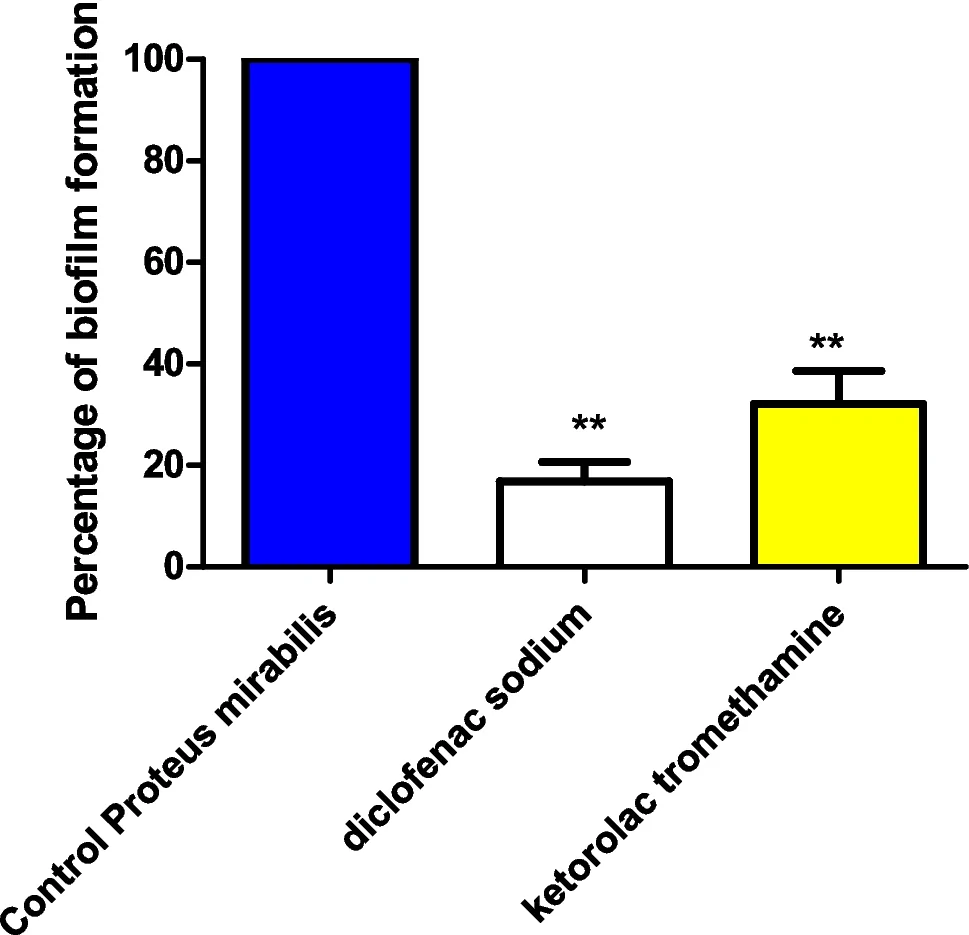
Diclofenac sodium and ketorolac tromethamine inhibited biofilm formation on catheter pieces**.** *P* < 0.05. **Significant inhibition

The biofilm formation on urinary catheter pieces in the presence and absence of the two drugs was assessed. Diclofenac sodium and ketorolac tromethamine showed good biofilm inhibiting activity. Diclofenac inhibited biofilm by 83.1%, while ketorolac tromethamine inhibited biofilm formation by 67.89% (Fig. 6).

#### Diclofenac sodium and ketorolac tromethamine reduced the expression of urease genes

**Fig. 7:**
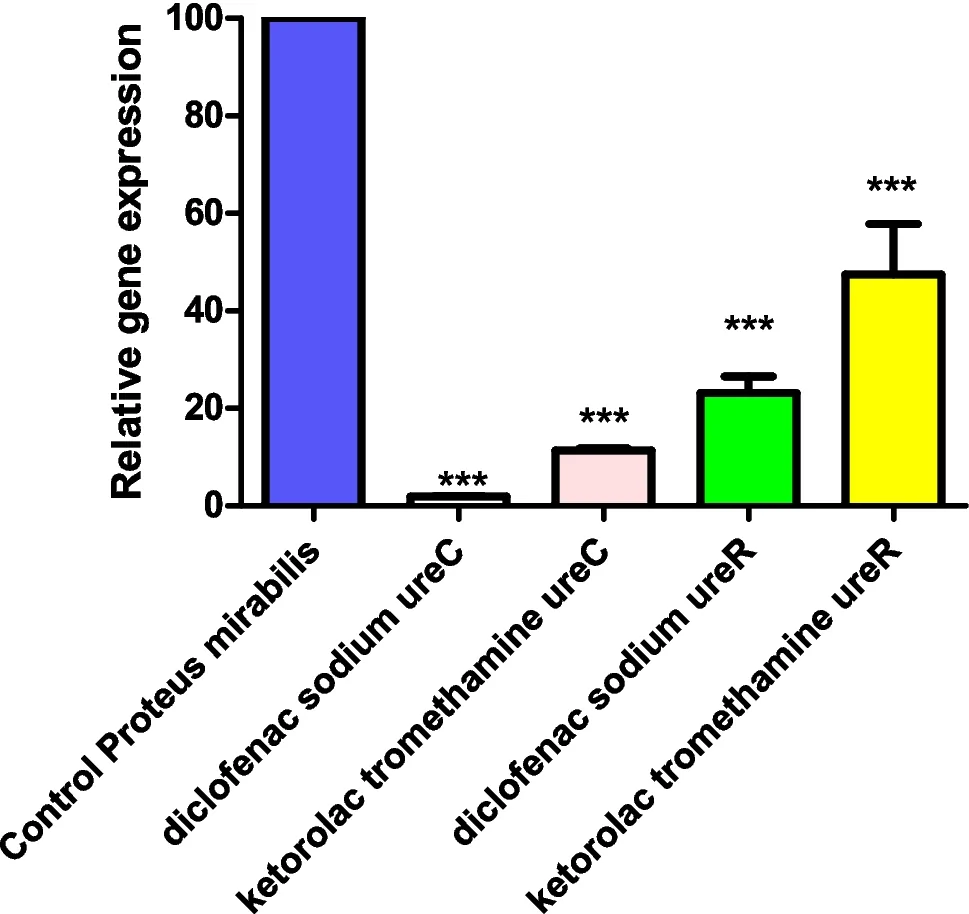
The effect of diclofenac sodium and ketorolac tromethamine on the expression of urease genes *ureC* and *ureR*. Diclofenac sodium and ketorolac tromethamine reduced the expression of *ureC* and *ureR. P* < 0.05. **Significant inhibition

To confirm the effects on urease activities by diclofenac sodium and ketorolac tromethamine, quantitative real-time PCR was performed in the presence and absence of both drugs. Diclofenac sodium and ketorolac tromethamine could downregulate urease genes *ureR* and *ureC*, but diclofenac sodium was more active (Fig. 7)*.* Diclofenac sodium reduced the expression of the *ureC* gene by 98.11% and *ureR* by 76.91%, while ketorolac tromethamine reduced the expression of the *ure*C gene by 88.61% and *ure*R by 52.52% (Fig. 7).

#### Diclofenac sodium and ketorolac tromethamine binding to urease enzyme

Molecular docking studies were conducted to further investigate the mechanism of urease inhibition by diclofenac sodium and ketorolac tromethamine. *Sporosarcina pasteurii* urease crystal structure (PDB: 4UBP; 1.55 Å) was utilized for the docking studies.

**Fig. 8:**
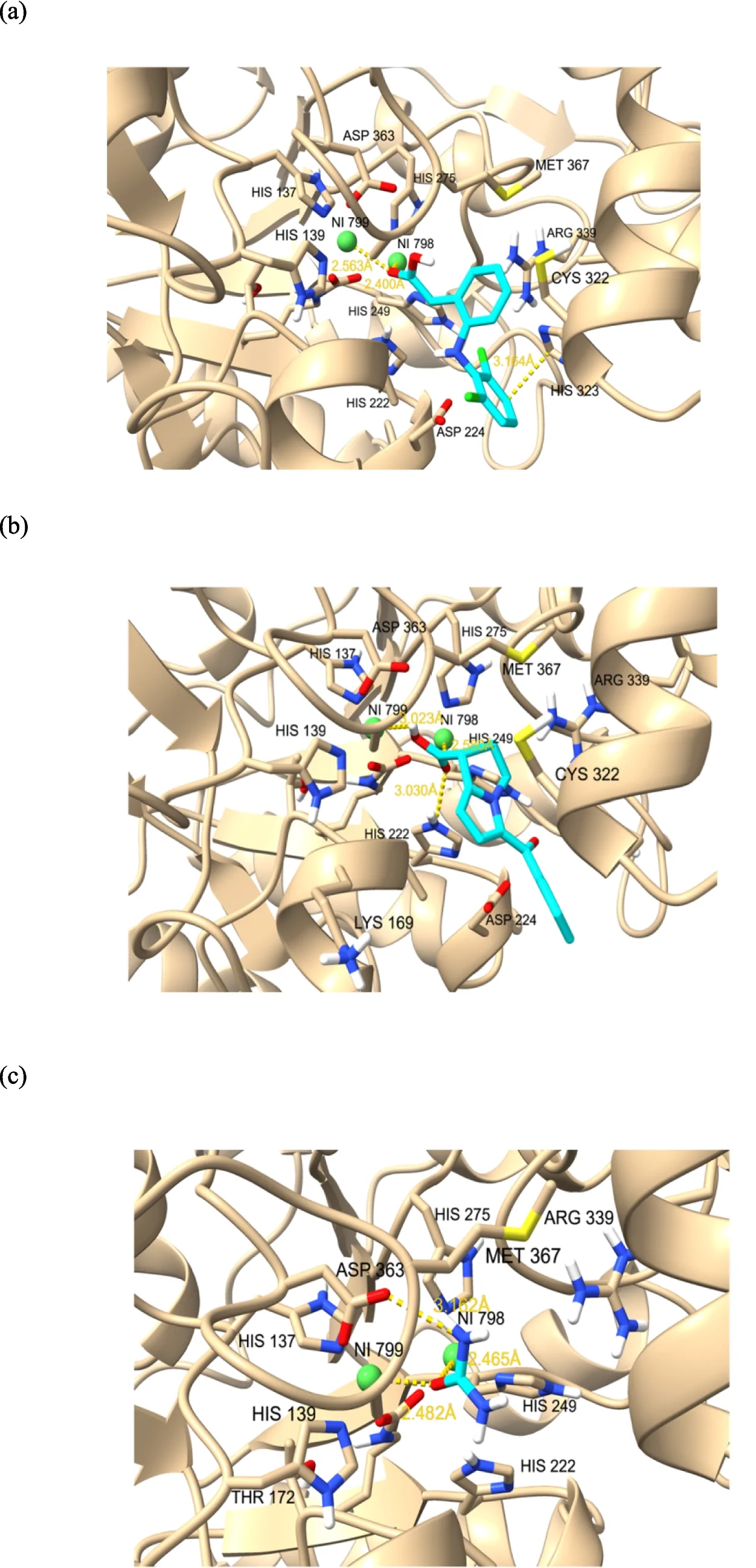
Three-dimensional ligand-enzyme interactions of (**a**) diclofenac (cyan sticks), (**b**) ketorolac (cyan sticks), and (**c**) urea (cyan sticks) within the active pocket of *S. pasteurii* urease

Diclofenac was able to fill the urease active pocket with a binding affinity of −6.4 kcal/mol. The carbonyl group of diclofenac coordinated with the two nickel ions with distances of 2.400 Å and 2.563 Å. On the other side, the dichlorophenyl ring of diclofenac formed an arene-arene interaction with His 323 (Fig. 8a). Ketorolac could fill the urease active pocket with a binding affinity of −6.3 kcal/mol. The carboxylic acid group of ketorolac coordinated with two nickel ions with distances of 2.580 Å and 3.023 Å. Additionally, the carbonyl group showed a hydrogen bond interaction with His 222 with a distance of 3.030 Å (Fig. 8b).

Urea, the natural substrate, was docked into the active pocket and showed good binding affinity (−5.1 kcal/mol). The carbonyl group of urea coordinated with the two nickel ions with distances of 2.465 Å and 2.482 Å. Moreover, the amino group formed hydrogen bond interactions with the acidic Asp 363 residue at a distance of 3.162 Å (Fig. 8c).

## Discussion

*Proteus mirabilis* secretes urease enzyme. Urease is a metalloenzyme that depends on nickel ion for activity. This enzyme is crucial for *Proteus mirabilis* because it enables it to cause catheter-associated urinary tract infections in addition to cystitis and pyelonephritis. The production of urease enzyme plays a role in crystalline biofilm formation in catheters that can lead to blocking of catheters and urine retention and even to severe complications such as septicemia and endotoxic shock **(**Pettersen et al. 2004; Trott and Olson 2010; Mobley 2019**).** Urease makes the urine pH alkaline. Alkaline pH provides suitable conditions to precipitate struvite and apatite crystals. The aggregation of apatite and struvite crystals leads to formation of urinary stones, to which bacteria adhere and remain viable within the layers of the crystals that represent a shield for protecting bacterial cells against antibiotics. Moreover, the ammonia produced by degradation of urea by urease enzyme is toxic to mammalian cells and may cause tissue damage due to high alkalinity. Furthermore, urease enables bacteria to evade immunity because urease contributes to chemotaxis and inhibits complement-mediated opsonization (Hu and Mobley 1990; Nakamura et al. 1998; Rokita et al. 1998; Schaffer and Pearson 2015; Wasfi et al. 2020; Yang et al. 2024).

In the light of the important roles of urease in the virulence of *Proteus mirabilis* causing CAUTIs, inhibition of urease is considered an important strategy to treat these infections. Inhibition of the urease enzyme can be accomplished by different mechanisms. One mechanism depends on the attachment of the inhibitor to nickel ions in the active site of urease, such as hydroxyurea. Another mechanism relies on the formation of covalent bonds that cover the enzyme center that has two nickel ions, leading to the inhibition of binding of urea with the enzyme, e.g., quinones. The third mechanism is the chelation of nickel ions necessary for enzyme activity, e.g., EDTA (Svane et al. 2020). Many natural compounds showed urease-inhibiting activities, such as polyphenols, flavonoids (Liu et al. 2020; Yener et al. 2020), alkaloids (Taha et al. 2020), triazole (Khan et al. 2020) thiadiazole, and coumarins (Alomari et al. 2019).

In this study, diclofenac sodium and ketorolac tromethamine were investigated for their capability to inhibit urease enzyme and crystalline biofilm formation on urinary catheters by *Proteus mirabilis.* The selection of these drugs was based on their previously reported metal chelating activity (Refat et al. 2013). We proposed that the drugs may potentially chelate nickel ions in the active site of urease enzyme and hence inhibit urease activity. To verify this hypothesis, a docking study was performed. Compared to urea, which is the natural substrate of urease enzyme, both diclofenac sodium and ketorolac tromethamine exhibited higher binding affinity to urease enzyme. Both drugs could coordinate with the two nickel ions of urease, indicating their potential inhibitory activities against *P. mirabilis* urease enzyme.

To confirm the results of the docking study, the in vitro activities of diclofenac sodium and ketorolac tromethamine against urease activity, pH increase, and crystalline biofilm formation were tested. Both drugs were used at sub-inhibitory concentrations (1/4 MIC) to avoid inhibition of bacterial growth that may affect urease activity, and the used concentrations showed no significant inhibitory effect on growth. This attribute was used in many previous studies (Abbas and Hegazy 2020; Askoura et al. 2020).

Diclofenac sodium and ketorolac tromethamine showed significant comparable inhibitory activities against urease activity in whole cells and in cell lysates. Moreover, the tested drugs could significantly reduce the increase in artificial urine pH and inhibit crystal formation in artificial urine. However, diclofenac sodium showed higher biofilm inhibiting activity than ketorolac tromethamine on urinary catheters. In previous reports, N-acetyl cysteine and dipropyl disulphide could inhibit urease activity and biofilm formation by *Proteus mirabilis* (Abdel-Baky et al. 2017). Diclofenac sodium and ketorolac tromethamine were more active as anti-biofilm agents than N-acetyl cysteine and dipropyl disulphide at ¼ MIC. On the other hand, N-acetyl cysteine and dipropyl disulphide showed higher urease inhibition than diclofenac sodium and ketorolac tromethamine. The ability of bacteria to produce biofilm increases its survivability under harsh external conditions. Moreover, biofilm-forming bacteria are highly resistant to antibiotics and to the host immune responses (Costerton 1999; Jacobsen and Shirtliff 2011; Al-Harthi et al. 2024).

In order to confirm the phenotypic results in our study, the inhibitory activities of diclofenac sodium and ketorolac tromethamine against urease-encoding genes *ureC* and *ureR* were assessed by quantitative real-time PCR. Significant downregulation of both genes was achieved with both drugs. However, diclofenac sodium could exert higher downregulation of urease genes. Urease gene cluster in *P. mirabilis* is composed of *ureABC* and *ureDEFG. ureABC* encodes the structural subunits of urease, while *ureDEFG* encodes the proteins that enhance the insertion of nickel ions into the active site of the urease enzyme (Mobley et al. 1995). The expression of the urease operon is activated by the protein UreR that binds to urea, so that the protein binds to *ureR* and *ureD* promoters, and RNA polymerase is recruited by UreR to activate transcription of urease (Thomas and Collins 1999; Poore et al. 2001; Gendlina et al. 2002). As the tested drugs could bind urease with higher affinity than urea, the binding of UreR protein to urea is inhibited, with subsequent interference with the activation of the urease operon.

## Conclusion

This study highlights the potential use of diclofenac sodium and ketorolac tromethamine as urease and biofilm inhibitors in *Proteus mirabilis*, suggesting their use in treating CAUTIs caused by *Proteus mirabilis.* However, in vivo tests and clinical trials are required.

## References

[CR1] Abbas HA (2015) Inhibition of virulence factors of *Pseudomonas aeruginosa* by diclofenac sodium. Roum Arch Microbiol Immunol 74(3–4):79–85 27328521

[CR2] Abbas HA, Hegazy WAH (2020) Repurposing anti-diabetic drug “sitagliptin” as a novel virulence attenuating agent in *Serratia marcescens*. PLoS ONE 15(4):e0231625. 10.1371/journal.pone.0231625 PMC7162429 32298346

[CR3] Abbas HA, Atallah H, El-Sayed MA, El-Ganiny AM (2020) Diclofenac mitigates virulence of multidrug-resistant *Staphylococcus aureus*. Arch Microbiol 202(10):2751–2760. 10.1007/s00203-020-01992-y 10.1007/s00203-020-01992-y 32737541

[CR4] Abdel-Baky RM, Ali MA, Abuo-Rahma GEDAA, AbdelAziz N (2017) Inhibition of urease enzyme production and some other virulence factors expression in *Proteus mirabilis* by N-acetyl cysteine and dipropyl disulphide. In: Donelli, G. (eds) Advances in Microbiology, Infectious Diseases and Public Health. Advances in Experimental Medicine and Biology, vol 973. Springer, Cham. 10.1007/5584_2016_197 28190143

[CR5] Al-Harthi HF, Eldrehmy EH, Afaf E, El-Hamid MIA (2024) Antimicrobial resistance and virulence genes profiles of *Staphylococcus aureus* in meat and meat products. Adv. Anim. Vet. Sci 12(7): 1290–1300. 10.17582/journal.aavs/204/12.7.1290.1300

[CR6] Alomari M, Taha M, Imran S, Jamil W, Selvaraj M, Uddin N, Rahim F (2019) Design, synthesis, *in vitro* evaluation, molecular docking and ADME properties studies of hybrid bis-coumarin with thiadiazole as a new inhibitor of Urease. Bioorg Chem 92:103235. 10.1016/j.bioorg.2019.103235 31494327

[CR7] Armbruster CE, Prenovost K, Mobley HL, Mody L (2017) How often do clinically diagnosed catheter-associated urinary tract infections in nursing homes meet standardized criteria? J Am Geriatr Soc 65(2):395–401. 10.1111/jgs.14533 PMC5310979 27858954

[CR8] Armbruster CE, Mobley HL, Pearson MM (2018) Pathogenesis of *Proteus mirabilis* infection. EcoSal Plus 8(1):10–1128. 10.1128/ecosalplus.ESP-0009-2017 PMC5880328 29424333

[CR9] Askoura M, Saleh M, Abbas H (2020) An innovative role for tenoxicam as a quorum sensing inhibitor in *Pseudomonas aeruginosa*. Arch Microbiol 202:555–565. 10.1007/s00203-019-01771-4 31732766

[CR10] Costerton JW (1999) Introduction to biofilm. Int J Antimicrob Agents 11:217–221 10.1016/s0924-8579(99)00018-7 10394973

[CR11] Durgadevi R, Veera Ravi A, Alexpandi R, Krishnan Swetha T, Abirami G, Vishnu S, Karutha Pandian S (2019) Virulence targeted inhibitory effect of linalool against the exclusive uropathogen *Proteus mirabilis*. Biofouling 35(5):508–525. 10.1080/08927014.2019.1619704 10.1080/08927014.2019.1619704 31144520

[CR12] Gendlina I, Gutman DM, Thomas V, Collins CM (2002) Urea-dependent signal transduction by the virulence regulator UreR. J Biol Chem 277:37349–37358 10.1074/jbc.M203462200 12147687

[CR13] Hooton TM, Bradley SF, Cardenas DD, Colgan R, Geerlings SE, Rice JC, Saint S,Schaeffer AJ, Tambayh PA, Tenke P, Nicolle LE (2010) Clin Infect Dis 10:321–324. 10.1086/650482 10.1086/650482 20175247

[CR14] Hu LT, Mobley HLT (1990) Purification and N-terminal analysis of urease from *Helicobacter pylori*. Infect Immun 58:992–998 10.1128/iai.58.4.992-998.1990 PMC258572 2318539

[CR15] Jacobsen SM, Shirtliff ME (2011) *Proteus mirabilis* biofilms and catheter-associated urinary tract infections. Virulence 2:460–465 10.4161/viru.2.5.17783 21921687

[CR16] Kafe FN, Abdullah EM, Selman IA (2023) Isolation and detection of *UreC* and *UreR* of *Proteus mirabilis* in gastrointestinal samples from patients with Crohn’s disease in Iraq. JPMS 12(4):37–42

[CR17] Khan I, Khan A, Halim SA, Saeed A, Mehsud S, Csuk R, Al-Harrasi A, Ibrar A (2020) Exploring biological efficacy of coumarin clubbed thiazolo [3, 2–b][1, 2, 4] triazoles as efficient inhibitors of urease: a biochemical and in silico approach. Int J Biol Macromol 142:345–354 10.1016/j.ijbiomac.2019.09.105 31593727

[CR18] Koch AL (1970) Turbidity measurements of bacterial cultures in some available commercial instruments. Anal Biochem 38(1):252–259 10.1016/0003-2697(70)90174-0 4920662

[CR19] Kovala-Demertzi D, Hadjikakou SK, Demertzis MA, Deligiannakis Y (1998) Metal ion–drug interactions. Preparation and properties of manganese (II), cobalt (II) and nickel (II) complexes of diclofenac with potentially interesting anti-inflammatory activity: behavior in the oxidation of 3, 5-di-tert-butyl-o-catechol. J Inorg Biochem 69(4):223–229. 10.1016/S0162-0134(97)10024-1

[CR20] Krajewska B (2009) Ureases I. Functional, catalytic and kinetic properties: a review. J Mol Catal B Enzym 59(1–3):9–21

[CR21] Li X, Zhao H, Lockatell CV, Drachenberg CB, Johnson DE, Mobley HLT (2002). Visualization of *Proteus mirabilis* within the matrix of urease-induced bladder stones during experimental urinary tract infection. Infect Immun 70:389–394. 10.1128/IAI.70.1.389-394.2002. 10.1128/IAI.70.1.389-394.2002 PMC127628 11748205

[CR22] Liu H, Wang Y, Lv M, Luo Y, Liu BM, Huang Y, Wang M, Wang J (2020) Flavonoid analogues as urease inhibitors: synthesis, biological evaluation, molecular docking studies and in-silico ADME evaluation. Bioorg Chem 105:104370. 10.1016/j.bioorg.2020.104370 10.1016/j.bioorg.2020.104370 33096309

[CR23] Livak KJ, Schmittgen TD (2001) Analysis of relative gene expression data using real-time quantitative PCR and the 2(-delta delta C(T)) method. Methods 25:402–408 10.1006/meth.2001.1262 11846609

[CR24] Milo S, Heylen RA, Glancy J, Williams GT, Patenall BL, Hathaway HJ, Thet NT, Allinson SL, Laabei M, Jenkins ATA (2021) A small-molecular inhibitor against *Proteus mirabilis* urease to treat catheter-associated urinary tract infections. Sci Rep 11(1):3726. 10.1038/s41598-021-83257-2 10.1038/s41598-021-83257-2 PMC7881204 33580163

[CR25] Mobley HLT (2019) Proteus mirabilis overview. Methods Mol Biol 2021:1–4. 10.1007/978-1-4939-9601-8_1 10.1007/978-1-4939-9601-8_1 31309490

[CR26] Mobley HL, Island MD, Hausinger RP (1995) Molecular biology of microbial ureases. Microbiol Rev 59:451–480 10.1128/mr.59.3.451-480.1995 PMC239369 7565414

[CR27] Modolo LV, de Souza AX, Horta LP, Araujo DP, de Fatima A (2015) An overview on the potential of natural products as ureases inhibitors: a review. J Adv Res 6(1):35–44. 10.1016/j.jare.2014.09.001 10.1016/j.jare.2014.09.001 PMC4293669 25685542

[CR28] Nakamura H, Yoshiyama H, Takeuchi H et al (1998) Urease plays an important role in the chemotactic motility of *Helicobacter pylori* in a viscous environment. Infect Immun 66:4832 10.1128/iai.66.10.4832-4837.1998 PMC108597 9746586

[CR29] Nicolle LE (2014) Catheter associated urinary tract infections. Antimicrob Resist Infect Control 3:23. 10.1186/2047-2994-3-23 10.1186/2047-2994-3-23 PMC4114799 25075308

[CR30] Niël-Weise BS, van den Broek PJ (2005) Urinary catheter policies for short-term bladder drainage in adults. Cochrane Database Syst Rev 3:CD004203.10.1002/14651858.CD004203.pub2. Update in: (2015). Cochrane Database Syst Rev 12:CD004203 10.1002/14651858.CD004203.pub2 16034924

[CR31] Pettersen EF, Goddard TD, Huang CC, Couch GS, Greenblatt DM, Meng EC, Ferrin TE (2004) UCSF chimera–a visualization system for exploratory research and analysis. J Comput Chem 25(13):1605–1612 10.1002/jcc.20084 15264254

[CR32] Poore CA, Coker C, Dattelbaum JD, Mobley HL (2001) Identification of the domains of UreR, an AraC-like transcriptional regulator of the urease gene cluster in *Proteus mirabilis*. J Bacteriol 183(15):4526–4535 10.1128/JB.183.15.4526-4535.2001 PMC95347 11443087

[CR33] Refat MS, Mohamed GG, Ibrahim MYS, Killa HMA, Fetooh H (2013) Synthesis and characterization of coordination behavior of diclofenac sodium drug toward Hg(II), Pb(II), and Sn(II) metal ions: chelation effect on their thermal stability and biological activity. Synth React Inorg, Met-Org, Nano-Met Chem 44(2):161–170. 10.1080/15533174.2012.752009

[CR34] Rokita E, Makristathis A, Presterl E et al (1998) *Helicobacter pylori* urease significantly reduces opsonization by human complement. J Infect Dis 178:1521–1525 10.1086/314459 9780279

[CR35] Schaffer JN, Pearson MM (2015) *Proteus mirabilis* and urinary tract infections. Microbiol Spectr. 10.1128/microbiolspec.UTI-0017-2013 PMC4638163 26542036

[CR36] Schaffer JN, Norsworthy AN, Sun TT, Pearson MM (2016) *Proteus mirabilis* fimbriae- and urease-dependent clusters assemble in an extracellular niche to initiate bladder stone formation. Proc Natl Acad Sci U S A 113(16):4494–4499. 10.1073/pnas.1601720113 PMC4843424 27044107

[CR37] Sekar A, Gil D, Tierney P, McCanne M, Daesety V, Trendafilova D, Muratoglu OK, Oral E (2024) Synergistic use of anti-inflammatory ketorolac and gentamicin to target staphylococcal biofilms. J Transl Med 22(1):102. 10.1186/s12967-024-04871-y 10.1186/s12967-024-04871-y PMC10809490 38273276

[CR38] Stepanovic S, Vukovic D, Dakic I, Savic B, Svabic-Vlahovic M (2000) A modified microtiter-plate test for quantification of staphylococcal biofilm formation. J Microbiol Methods 40(2):175–179. 10.1016/s0167-7012(00)00122-6 10699673

[CR39] Stickler DJ (2014) Clinical complications of urinary catheters caused by crystalline biofilms: something needs to be done. J Intern Med 276(2):120–129. 10.1111/joim.12220 10.1111/joim.12220 24635559

[CR40] Stickler DJ, Feneley RCL (2010) The encrustation and blockage of long-term indwelling bladder catheters: a way forward in prevention and control. Spinal Cord 48(11):784–790. 10.1038/sc.2010.32 10.1038/sc.2010.32 20368711

[CR41] Svane S, Sigurdarson JJ, Finkenwirth F, Eitinger T, Karring H (2020) Inhibition of urease activity by different compounds provides insight into the modulation and association of bacterial nickel import and ureolysis. Sci Rep 10(1):8503. 10.1038/s41598-020-65107-9 10.1038/s41598-020-65107-9 PMC7244745 32444844

[CR42] Taha M, Rahim F, Khan AA, Anouar EH, Ahmed N, Shah SAA, Ibrahim M, Zakari ZA (2020) Synthesis of diindolylmethane (DIM) bearing thiadiazole derivatives as a potent urease inhibitor. Sci Rep 10:7969 10.1038/s41598-020-64729-3 PMC7224224 32409737

[CR43] Tanaka T, Kawase M, Tani S (2003) Urease inhibitory activity of simple alpha, beta-unsaturated ketones. Life Sci 73(23):2985–2990. 10.1016/s0024-3205(03)00708-2 14519447

[CR44] Thomas VJ, Collins CM (1999) Identification of UreR binding sites in the *Enterobacteriaceae* plasmid-encoded and *Proteus mirabilis* urease gene operons. Mol Microbiol 31(5):1417–1428 10.1046/j.1365-2958.1999.01283.x 10200962

[CR45] Trott O, Olson AJ (2010) AutoDock Vina: improving the speed and accuracy of docking with a new scoring function, efficient optimization, and multithreading. J Comput Chem 31(2):455–61. 10.1002/jcc.21334. http://europepmc.org/abstract/MED/19499576 10.1002/jcc.21334 PMC3041641 19499576

[CR46] Wasfi R, Hamed SM, Amer MA, Fahmy LI (2020) *Proteus mirabilis* biofilm: development and therapeutic strategies. Front Cell Infect Microbiol 10:414 10.3389/fcimb.2020.00414 PMC7456845 32923408

[CR47] Wayne P (2011) Clinical and laboratory standards institute. Performance standards for antimicrobial susceptibility testing. Inform Suppl 31(1):100–121

[CR48] Yang A, Tian Y, Li X (2024) Unveiling the hidden arsenal: new insights into *Proteus mirabilis* virulence in UTIs. Front Cell Infect Microbiol 14:1465460 10.3389/fcimb.2024.1465460 PMC11599158 39606746

[CR49] Yener I, Kocakaya SO, Ertas A, Erhan B, Kaplaner E, Oral EV, Yilmaz-Ozden T, Yilmaz MA, Ozturk M, Kolak U (2020) Selective *in vitro* and *in silico* enzymes inhibitory activities of phenolic acids and flavonoids of food plants: relations with oxidative stress. Food Chem 327:127045. 10.1016/j.foodchem.2020.127045 32464460

[CR50] Zhang W, Niu Z, Yin K, Liu P, Chen L (2013) Quick identification and quantification of *Proteus mirabilis* by polymerase chain reaction (PCR) assays. Ann Microbiol 63:683–689. 10.1007/s13213-012-0520-x

